# Parasitic sea louse infestations on wild sea trout: separating the roles of fish farms and temperature

**DOI:** 10.1186/s13071-018-3189-6

**Published:** 2018-11-29

**Authors:** Knut W. Vollset, Lars Qviller, Bjørnar Skår, Bjørn T. Barlaup, Ian Dohoo

**Affiliations:** 1 Uni Research Environment, LFI - Freshwater Biology, Nygårdsporten 112, 5006 Bergen, Norway; 2 Norwegian Veterinary Institute, P.O. Box 750, Sentrum, N-0106 Oslo, Norway; 3 Department of Health Management, University of PEI, Charlottetown, PEI C1A 4P3 Canada

**Keywords:** Aquaculture, Brown trout, Climate, *Lepeophtheirus salmonis*, Sea lice

## Abstract

**Background:**

The causal relation between parasitic sea lice on fish farms and sea lice on wild fish is a controversial subject. A specific scientific debate has been whether the statistical association between infestation pressure (*IP*) from fish farms and the number of parasites observed on wild sea trout emerges purely because of a confounding and direct effect of temperature (*T*).

**Methods:**

We studied the associations between louse infestation on wild sea trout, fish farm activity and temperature in an area that practices coordinated fallowing in Nordhordland, Norway. The data were sampled between 2009 and 2016. We used negative binomial models and mediation analysis to determine to what degree the effect of *T* is mediated through the *IP* from fish farms.

**Results:**

The number of attached lice on sea trout increased with the *T* when the *IP* from fish farms was high but not when the *IP* was low. In addition, nearly all of the effect of rising *T* was indirect and mediated through the *IP*. Attached lice remained low when neighbouring farms were in the first year of the production cycle but rose substantially during the second year. In contrast to attached lice, mobile lice were generally seen in higher numbers at lower water temperatures. Temperature had an indirect positive effect on mobile louse counts by increasing the *IP* which, in turn, raised the sea trout louse counts. Mobile louse counts rose steadily during the year when neighbouring farms were in the first year of the production cycle and stayed high throughout the second year.

**Conclusions:**

The estimates of the *IP* effect on louse counts along with the clear biennial pattern emerging due to the production cycle of fish farms clearly indicate that fish farms play an important role in the epidemiology of sea lice on wild sea trout. Furthermore, the mediation analysis demonstrates that a large proportion of the effect of *T* on louse counts is mediated through *IP*.

**Electronic supplementary material:**

The online version of this article (10.1186/s13071-018-3189-6) contains supplementary material, which is available to authorized users.

## Background

Human activities can dramatically alter natural disease and parasite dynamics in wild animals [1, 2]. On land, such dynamics are often relatively easy to understand due to the structural proximity to humans [3]. In the marine habitat, the difficulties of appropriate surveillance in combination with the vastness of the ocean have made it more difficult to infer causal relations between human activities and observed disease or parasite dynamics in wildlife [4]. Sea lice (*Lepeophtheirus salmonis* and *Caligus* spp.) are crustacean ectoparasites that have been at the core of a long-running debate regarding the impact of intensive aquaculture on wild salmonids [5]. The main concern relates to (i) the decoupling of parasite abundance and host density when additional parasites produced in fish farms spillover to wild fish [6]; (ii) the potential interference with the natural allopatric habitat use of adult and migrating salmon smoults [7]; and (iii) the evolutionary changes in virulence induced by the presence of fish farms [8, 9].

For Atlantic salmon, impacts on their physiology and survival attributable to lice have been documented through a series of laboratory studies. These studies have shown that louse abundances above 0.1 louse per gram of fish weight on post-smoults (i.e. young fish that have recently entered the ocean) leads to physiological imbalance and increased stress hormones, whereas high loads (> 0.3 louse per gram of fish) can lead to acute death [10–12]. Consequently, monitoring sea lice on sea trout has become an important (and time-consuming) management task in countries harbouring native wild salmonids and with salmonid fish farming [13, 14]. Surveillance programmes have shown that these above-threshold levels are frequently observed on wild salmonids in areas with a high abundance of net pen fish farms [15, 16], but are more seldom observed in areas without fish farms [17, 18]. However, the role of fish farms as the source of infestation is still controversial.

Coordinated fallowing of fish farms has been applied extensively in Norway and internationally as a mitigation tool to limit the spread of diseases that spread horizontally from farm to farm. The underlying idea of the coordinated fallowing is to break the life-cycle of parasites and diseases that proliferate in high-host-density conditions [19]. In the case of exotic diseases or diseases with low transmission rates, fallowing is used to eradicate the disease locally and reduce the risk of epidemic outbreaks. If the disease or parasite is common or endemic to the surrounding environment and/or the transmission rate is relatively high (all of which are true for sea lice), fallowing is used to limit population growth, limit the use of chemical treatment and/or combat the spread of strains resistant to chemical treatment. Atlantic salmon usually require between 16 and 24 months from the time post-smoults are stocked in the net pens to reach market size [20]. Consequently, even though fallowing lowers louse levels during the first year of production, several studies have demonstrated that louse levels increase and usually peak sometime during the second year of production [21–23]. This general pattern is most likely driven by a combination of a high population growth rate, a high rate of self-infestation, and external infestation from nearby farms [24, 25]. This biennial pattern in fish farms creates an ideal field experiment to test the causal relation between sea lice on wild fish and spillover effects from nearby fish farms. Several studies have shown that louse infestation on wild fish is higher when nearby fish farms are in their second year of production [22, 26–28].

Recently, studies that correlate the estimated infestation pressure by sea lice with farms with louse counts on wild fish have become more common. For example, Serra-Llinares et al. [29] and Helland et al. [30] used various statistical methods to link the two and argued that the significant correlation was proof that lice from fish farms directly infested wild fish in the vicinity of the fish farm. Such associations, however, are not without controversy. For example, Jansen et al. [31] argued that the modelled impact of fish farming on sea trout conducted by Serra-Llinares et al. [29] did not consider the seasonal progression of sea louse infestation on wild and farmed fish and argued that the correlation between the two could potentially occur as a consequence of temperature. In response, the authors re-analysed their data, showing that louse infestation pressure from fish farms was an important factor even after correcting for temperature [32]. However, although they corrected for temperature in their analysis, the high collinearity between the variables and the lack of data covering the seasonal progression, allowed the question of cause and effect to linger.

In this study, we revisit this contentious issue by exploring an independent dataset derived from sea louse surveillance on sea trout between April and August in the years 2009–2016 in the region of Nordhordland, on the west coast of Norway. The surrounding fish farms in the region conducted coordinated fallowing throughout the sampling period, thus creating an ideal time series for investigating the role of fish farming on the sea louse infestations on sea trout in marine waters. From 2012 to 2016, data were available from surrounding fish farms to estimate infestation pressure (*IP*) and temperature (*T*). We applied negative binomial generalized linear models (hereafter called ‘negative binomial models’) to these two datasets to study the effect of fish farms on louse counts on wild sea trout.

Our second goal was to attempt to apply a mediation analysis to understand the link between temperature and *IP* from fish farms. Mediation analysis is a statistical procedure designed to determine how much of the total effect of a variable (e.g. smoking) on an outcome (e.g. risk of a heart attack) is an indirect effect due to the effect of smoking on a mediating variable (e.g. blood pressure) and how much is a direct effect (i.e. the increased risk even if blood pressure is not raised) [33]. If there is interaction between the effects of smoking and blood pressure (e.g. smoking is more detrimental in someone with high blood pressure than in someone with low blood pressure), mediation further separates the effect of smoking into ‘pure’ effects (ignoring the effect of smoking on blood pressure) and ‘total’ effects (accounting for the effect of smoking on blood pressure). In this specific case, we apply a mediation analysis in an attempt to determine the degree to which the effects of annual variation and water temperature on louse counts on wild sea trout is mediated by louse infestation pressure from fish farms.

## Methods

### Description of sampling area

**Fig. 1:**
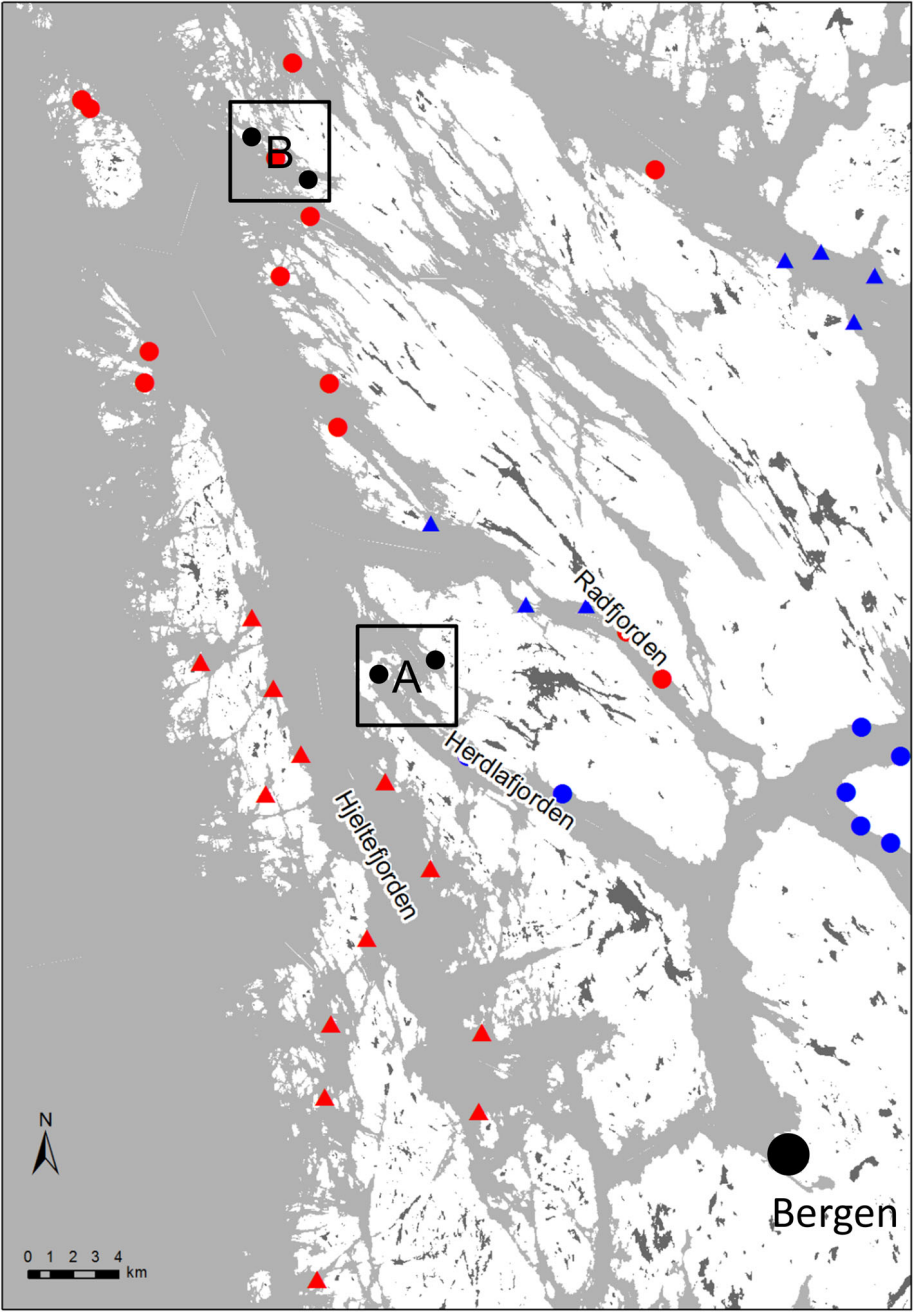
Map of the sampling area. The locations of the city of Bergen and the fish farms are indicated on the map (A, Area A; B, Area B). The different colours and shapes represent the following: red triangles, fish farms fallowing in spring in odd years; red circles, fish farms fallowing in autumn in even years; blue triangles, fish farms fallowing in spring in even years; red circles, fish farms fallowing in autumn in odd years. Information provided by the Norwegian Food Authority. Black circles within each area are the exact locations of the sampling sites

The present study was conducted on the west coast of Norway, north of the city Bergen (Fig. 1). The study area is a complex fjord system. It is located in the outer part of the migration route of several salmon and sea trout populations habiting rivers running into the fjord Osterfjorden, one of which is the famous Vosso River [34]. The inner fjord system (not depicted on the map) spills into the outer fjord system at a narrow pass (left hand side of map) and then splits into three fjord arms (Hjeltefjorden, Herdlafjorden and Radfjorden) that eventually form a rugged archipelago. The topography and bathymetry of the inner fjord results in water in which the upper level has low salinity (< 20 ppt) during spring (except during abnormal weather events). Consequently, migrating salmonids encounter sea lice (which do not tolerate low salinity) mostly during the migration in the outer fjord [35]. This outer fjord is where sea louse monitoring has been conducted.

### Sea louse monitoring

The number of sea lice on wild sea trout (N_lice_) was monitored between 2009 and 2016. The sampling was done using either specially constructed trap nets [36] or was taken from by-catch in gillnets. Sea lice on wild fish were monitored during spring and early summer because this is believed to be the most sensitive recruitment period for salmon and sea trout [37]. Sampling by trap net was done on a dedicated location (Fig. 1, Area A) at Herdla. This location was monitored using one, two or three trap nets every year from 2010 to 2016. The first year (2009), before the trap net design was available, gillnets were used. Sampling of bycatch during deployment of gillnets was done by one dedicated fisher north of Radøy (Fig. 1, Area B). The trap nets were checked once a day when catches were low and twice a day when catches were high. Sea trout were normally sampled in four periods ranging from 2 to 10 days, where the goal was to catch approximately 30 trout. The sampling period dates varied somewhat with years due to logistics and catchability. Sampling from Area B was done whenever the fisher deployed the net, and the dataset from this site is therefore unbalanced. In sampling Area A, large individual fish (> approx. 1 kg) were released with minimum handling and without being surveyed for sea lice. However, some larger individuals were euthanized and sampled according to defined humane endpoints, as required by the animal welfare protocol. All sampled fish were euthanized with a blow to the head and placed in individual plastic bags and frozen for later inspection of the sea lice.

Fish and associated sea lice were thawed and then inspected with a lens in the laboratory. Care was taken to analyse whether any lice were left in the plastic bag. Lice were identified to their developmental stage according to Hamre et al. [38]. Other fish measurements recorded were weight (in grams), total length (in mm), fin erosion, other damages and scale loss. All fish with a scale loss above 50% were excluded from further analysis. For the purpose of the analysis explained below, we grouped the louse developmental stages in ‘attached’ (copepodite and chalimus) and ‘mobile’ (preadult and adult) stages.

### Infestation pressure from fish farms

The infestation pressure (*IP*) produced in the surrounding fish farms was calculated for the two sampling sites as explained in Kristoffersen et al. [25], using life history estimates from Stien et al. [39] and a distance function from Aldrin et al. [24]. In short, the *IP* is based on the distance penalized sea lice reproduction from the surrounding fish farms, which include the mortality during the development from hatching of the eggs to the attached and mobile sea lice. The infestation pressures were also calculated for some time prior to the observations, as a function of sea lice development time. A condensed description from the description in Kristoffersen et al. [25] follows below.

Every active fish farm is obliged to report the fish abundance and average number of adult female sea lice per fish every week to the responsible authorities. The total abundance of reproducing salmon lice (*n*_*AF*_) in the fish farm is the product of these two factors. The fecundity (*F*) in the model was defined as the temperature-dependent daily reproduction from one adult female sea louse, according to the following formula:

*F* = 300 eggs/[41.98/[*T* − 10 + (41.98 × 0.338)]]^2^

where 300 eggs are the mean combined reproduction in both egg strings, the denominator in the fraction is the development time of the egg strings, and *T* is the temperature (°C). Total sea louse reproduction in a farm *i* at time *t* can be expressed by *F*_*i*_ *× n*_*AF(i)*_. The reproduction was calculated daily.

The infestation pressure used herein assumes that the larval concentration decreases with increasing seaway distance to the release from the salmon farms, following the relative risk function in Kristoffersen et al. [25]:

RR_*ij*_ = exp(−1.444 − ((d_*li*_)^0.57^ − 1)/0.57)/ exp(−1.444 − ((d_*ii*_)^0.57^ − 1)/0.57)

where RR_*li*_ is the relative risk of infestation to location *l* from farm *i* based on the seaway distance to the farm and the distance from the farm to itself (d_*ii*_ = 0) [24].

The seaway distance is a measure of the shortest way in the water around islands and obstacles, calculated with the *gdistance* package in R, using 16 directions [40]. The total infestation pressure experienced in location *l* is the sum of the infestation pressure from all active farms within 200 km.

In this study, we compare the infestation pressure with observed infestation on wild fish. We must therefore account for a time delay, which is the number of days from the larval hatch until the lice appear on the fish. The time delay during the pelagic stage is a function of the temperature-dependent development time during the nauplii stages (35 degree-days) and 4 days until attachment is successful. The latter parameter is based on the average survival during the copepodite stage. The daily mortality rate *q* is a constant set to 0.17 in the nauplii stages. Time-dependent mortality in the planktonic stages therefore depends on the time delay from the hatching of the eggs until attachment. The above calculations accounts for the time delay and mortality until the attached stage of sea lice. To get to the mobile lice stage, we must also account for the development time (155 degree-days) and the constant daily mortality rate (0.05) during the entire period of attachment.

### Temperature data

The estimated temperature (*T*) at a given place and time is the distance-weighted average temperature measured in surrounding fish farms, according to the following formula:

$$ T=\sum \limits_i^n{T}_i\left(\left({Dist}_{Max}-{Dist}_i\right)/\sum Dist\right) $$

where *i* is the indicated fish farm, counting from 1 to the number of active fish farms in Norway; *T*_*i*_ the temperature measured in farm *i*, (*Dist*_*Max*_ - *Dist*_*i*_) is the inverse seaway distance from the point of interest to farm *i*, and the denominator, Σ*Dist* is the sum of all seaway distances. As with the louse counts on fish farms, the temperature was only available from 2012 and onwards. Since we do not have any data on where the fish that were caught had been prior to sampling, we opted to calculate both *T* and *IP* for the sampling locations.

### Production zones and cycles

Coordinated fallowing was employed in the study area during the entire study period (2009–2016). The fallowing zones are not obligatory, but are based on a consensus between companies of fish farmers and the food authorities. The fallowing zones were a compromise between what was logistically possible and creating zones large enough to be effective in reducing disease spread. Fish farms that could influence wild sea trout in Area A mainly fallowed during autumn of odd years, whereas fish farms influencing Area B mainly fallowed in the spring of even years. Given the general pattern of highest louse counts in fish farms during the second year of a production cycle, even years were expected to show the highest infestation pressure of sea lice from fish farms on our sampled fish. The reported louse numbers from fish farms are publicly available online (https://www.barentswatch.no/).

### Data analysis

**Fig. 2:**
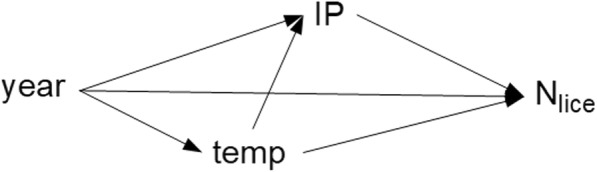
Postulated causal diagram showing relations among the *Year*, water temperature (*T*), estimated infection pressure arising from adjacent salmon farms (*IP*), and attached or mobile louse counts on wild sea trout (N_lice_)

As explained above, the *IP* estimates were only available from 2012 to 2016. Consequently, we conducted analyses on two sets of data. Dataset 1 consisted of data with *IP* estimates collected between 2012 and 2016. Dataset 2 contained the more limited data (with no *IP* estimates) that covered the longer period (2009–2016). In Dataset 1, data on 428 fish were available, but four sea trout with abnormally short lengths (< 5 cm) or long lengths (> 100 cm) were assumed to have incorrect data recorded and were excluded, leaving 424 fish with complete data. The factors of interest that we hypothesized to have affected counts of attached (N_liceA_) and mobile (N_liceM_) lice were: *Year*, *T* and *IP*. A causal diagram showing our hypothesized relations among *Year*, *T*, *IP* and N_lice_ is shown in Fig. 2. In Dataset 2, where the *IP* was missing from 2009–2011, the sampling day-of-year served as a proxy measure of louse exposure because of the strong relation between date and exposure; complete data were available for 1051 fish. Attached louse counts (N_liceA_) (copepodites and chalimus) and mobile lice (N_liceM_) (preadult and adult) were analysed separately in both data sets.

#### Dataset 1: Attached louse counts (N_liceA_)

##### Data manipulations and descriptive statistics

**Fig. 3:**
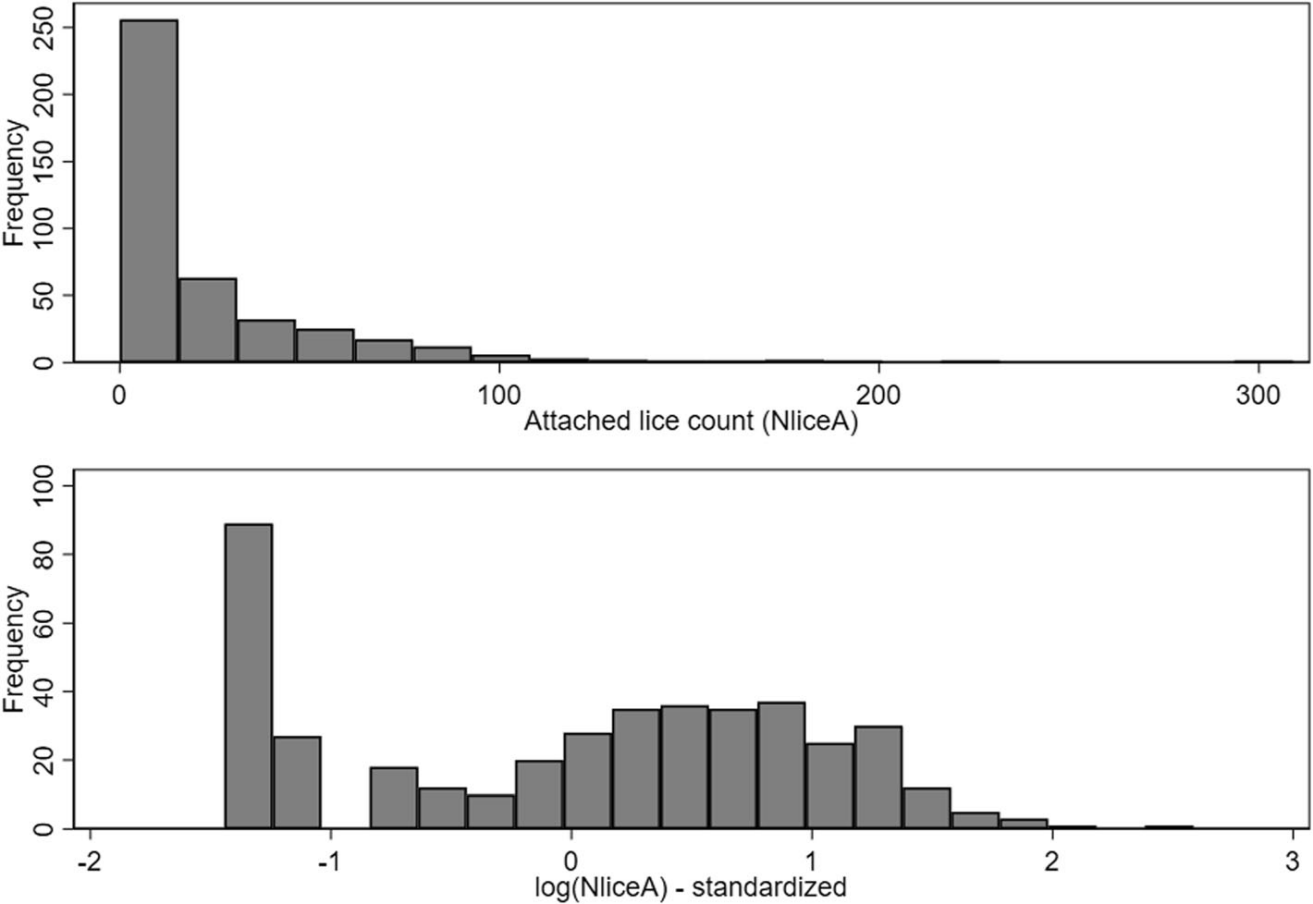
Histograms of attached louse counts (N_liceA_) and standardized version of log(N_liceA_) from Dataset 1. *Note*: one fish with attached count of 770 was excluded from top graph for scaling reasons

The exposure estimate (*IP*) was log-transformed, as were N_liceA_ and N_liceM_ (after first adding 0.5 to all fish which had counts of zero). Subsequently, all continuous variables were standardized by subtracting their mean value and dividing by their standard deviation. A list of the variables of interest is presented in Table 1, along with the descriptive statistics of the variables. Histograms of the N_liceA_ and log(N_liceA_) were generated (Fig. 3). The linearity of all relations among *T*, *IP* and log(N_liceA_) were evaluated using Lowess smoothed curves. Where evidence of nonlinearity was observed, quadratic terms were added to the regression models and retained if statistically significant.

**Table 1:** Descriptive statistics of variables of interest in datasets 1 and 2

Variable	Acronym^a^	Dataset 1			Dataset 2		
		*n*	Mean ± SD	Range	*n*	Mean ± SD	Range
Attached lice count	N_liceA_	424	24.73 ± 50.46	0–770	1051	17.3 ± 38.4	0–770
Log (attached lice count) - standardized	N_liceA_std_	424	0.01 ± 1.00	-1.44–2.59	1051	0.0 ± 1.0	-1.3–2.98
Mobile (adult) lice count	N_liceM_	424	11.79 ± 16.8	0–105	1051	15.3 ± 23.2	0–249
Log (mobile lice count) - standardized	N_liceM_std_	424	0.00 ± 1.00	-1.42–2.06	1051	0.0 ± 1.0	-1.43–2.36
Temperature °C		424	9.83 ± 1.99	5.96–13.84	na	na	na
Temperature - standardized	*T*	424	0.00 ± 1.00	-1.933–2.004	na	na	na
Estimate lice exposure		424	4,884,096 ± 6,660,133	10,060–3.77×10^7^	na	na	na
Log (estimate lice exposure) - standardized	*IP*	424	14.03 ± 2.10	9.22–17.44	na	na	na
Day		na	na	na	1051	158.0 ± 19.2	95–214

^a^Acronym used throughout the manuscript and in all tables

*Abbreviations*: *n* number of observations, *log* log-transformed variable using the natural logarithm, *SD* standard deviation, *na* not available, *N*_*liceA*_ number of attached stages of lice, *N*_*liceM*_ number of adult or mobile stages of lice, *T*, standardized water temperatures described in the text, *IP* estimated infestation pressure described in the text

##### Evaluation of year as a confounder

The year was identified as a potential confounder of the effects of both *T* and *IP*. To determine the strength of the association among the *Year* and *T* and *IP*, these values were regressed on *Year* (converted to a set of indicator variables), and the *R*^2^ of the regression was noted. A very high *R*^2^ for *IP* regressed on *Year* suggested that collinearity might prevent the inclusion of year as a confounder in any models incorporating *IP*. Indeed, including the year in a model with *IP* completely eliminated any *IP* effects. Consequently, *Year* was excluded from the negative binomial models. See also Additional file 1: Text 1 and Figure S1, for a more detailed discussion on this.

##### Negative binomial models

Negative binomial (NB) and zero-inflated negative binomial (ZINB) models were fitted to determine how *T* and *IP* (and their interaction) influenced the N_liceA_. A ZINB accounts for the possibility that the number of zero counts observed was higher than would be expected from the corresponding NB model. In a ZINB, zero counts are assumed to arise in one of two ways, either as very low values from the negative binomial distribution or through some other mechanism (e.g. trout not exposed to any lice).

In general, model building was achieved by using a combination of criteria for selecting the best model. These included using AIC values for selecting the model type (e.g. fixed or random effects for the year in Dataset 2) and using *P*-values and AIC for determining if the interaction and quadratic effect terms should be retained in models, with parameter estimates ultimately being evaluated as to whether they were reasonable. Terms that were not significant (*P* < 0.05) and not a component of a significant interaction term were removed. The Akaike information criterion (AIC) values of the final NB and ZINB models were compared, and a likelihood ratio test was performed to compare the two types of models.

##### Mediation analyses

Mediation analysis software that allows for an NB or ZINB model of the outcome of interest is not available, so mediation analyses with log(N_liceA_) as the outcome, *Year* or *T* as the exposure of interest, and either *T* or *IP* as the mediators were carried out. To determine whether a linear (Guassian) model for log(N_liceA_) was a reasonable approximation for a ZINB model of N_liceA_, we compared the coefficients from the models (both are on the log(N_liceA_) scale) and determined the correlation between observed values of louse counts with those predicted from the two models on both the log and count scales (Additional file 1: Text 2, Table S1 and S2).

Initially, mediation analysis was used to determine what portion of the effects of *T* were mediated through *IP*. This included consideration of the possibility of a T*IP interaction. Subsequently, mediation analysis with *Year* as the exposure of interest was carried out. Because *Year* was a 5-level categorical variable (2012–2016), the level with the lowest louse counts (2013) was selected as the baseline so that all year effects estimated would be positive. Either *T* or *IP* was considered the mediator and interaction effects between *Year*, and either of the mediators (*T* or *IP*) were examined. In all analyses, the quadratic effects of the mediators were included if required. Output derived from the mediation analyses consisted of estimates of direct and indirect effects and % mediated.

#### Dataset 1: Adult (mobile) louse counts (N_liceM_)

The regression model building processes described above were repeated for the outcome N_liceM_, with the exception that inclusion of *Year* as a confounder in the negative binomial models of N_liceM_ did not have a deleterious effect on the estimation of *IP* effects, so *Year* was retained as a random effect in those regression models. As with N_liceA_, marginal estimates of N_liceM_ were plotted against *IP* at each of three water temperatures (7, 10 and 13 °C). In mediation analyses with *Year* as the exposure of interest, 2014 was selected as the baseline (reference) year because it had the lowest adult louse counts.

#### Dataset 2: Attached and adult (mobile) louse counts (N_liceA_ and N_liceM_)

The possibility of using sampling day-of-year (*Day*) as a proxy for *IP* in Dataset 2 was evaluated using data from Dataset 1 in which both *IP* and day were recorded. Scatter plots and Lowess smoothed curves for *IP vs Day* were generated for each year. Separate plots were generated for years in which the regional aquaculture operations were in their first and second year of production, i.e. production cycle (*PC*). Correlations within years ranged from 85 to 98%. Plots and correlations are presented in Additional file 1: Table S1 and Figure S2). We concluded that *Day* was an acceptable surrogate for *IP*. As with the predictors in Dataset 1, *Day* was standardized to have a mean of zero and standard deviation of one.

As with Dataset 1, descriptive statistics were computed (Table 1) and the linearity of continuous predictors (in this case only *Day*) evaluated using Lowess smoothed curves and polynomial functions as necessary. Both negative binomial (NB) and zero-inflated negative binomial (ZINB) models were fitted to N_liceA_ and N_liceM_ with the ZINB model retained if it was statistically superior to the NB model based on a likelihood ratio test. In all cases, year of the production cycle (*PC* - first *vs* second) and *Year* were retained as possible confounders. An interaction term between *Day* and *PC* was included.

All basic analyses and negative binomial models were fitted using Stata (version 15). Mediation analyses were carried out using add-on programmes - medeff in Stata and mdeflex in R [41]. Unless otherwise noted, statistical significance was evaluated at *P* = 0.05.

## Results

### Dataset 1: Attached louse counts (N_liceA_)

#### Causal diagram and descriptive statistics

The causal diagram of the variables of interest is shown in Fig. 2. Descriptive statistics of the key variables of interest are presented in Table 1. Histograms of the N_liceA_ (with an extreme value of 770 removed for scaling purposes) and the log(N_liceA_) are shown in Fig. 3. The Lowess smoothed curves of all the relations among the standardized versions of the continuous variables (including the T*IP interaction) are shown in Additional file 1: Figure S3. Evidence exists of non-linearity in the effects of *IP*, so a quadratic term was added to an unconditional NB model and found to be significant (*P* = 0.02). Consequently, the quadratic term was retained in all subsequent models.

#### Negative binomial models

As noted above, the quadratic term for *IP* was significant, so it was retained in all subsequent models. The interaction between *T* and the linear component of *IP* was statistically significant and, consequently, was included in all models. The interaction with the quadratic component of *IP* was not significant (*P* = 0.22) and, hence, was not retained. Only the linear component of *IP* was significant in the logistic portion of ZINB models, so neither the *T* nor the T*IP interaction were included in this part of the model.

The AIC of the final ZINB model was smaller than that of the corresponding NB model, indicating that the ZINB model was preferred. The likelihood ratio test comparing the two models was highly significant (*P* < 0.001).

**Fig. 4:**
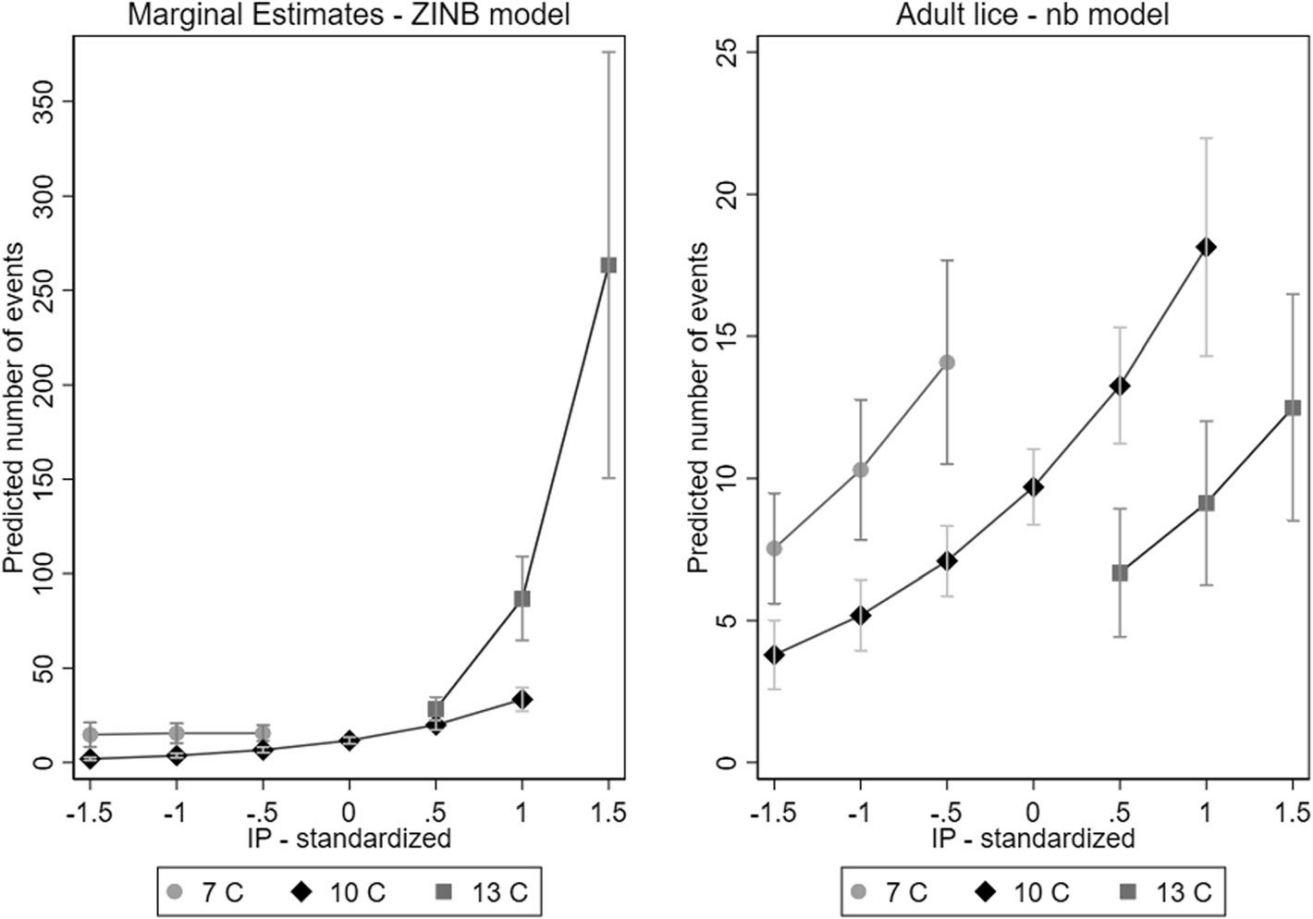
Predicted values from a zero-inflated negative binomial model of N_liceA_ and a negative binomial model of N_liceM_. Lines represent the predicted values at water temperatures of 7 °C, 10 °C and 13 °C. Lines only cover the range of *IP* which existed at the relevant water temperature

The final ZINB model is presented in Table 2. The estimate of alpha (dispersion parameter) for the NB portion of the ZINB model was 1.07 (95% CI, 0.88–1.31), indicating the variance in N_liceA_ was much greater than would have been expected under a Poisson distribution. The main effects of *IP* (linear and quadratic) and T*IP interaction were highly significant. Plots of marginal estimates of louse counts (N_liceA_) *vs* exposure (*IP*) were generated for each of three water temperatures (7 °C, 10 °C and 13 °C, representing approximately the 10th, 50th and 90th percentiles of *T*, Fig. 4). The plot of marginal estimates (Fig. 4, left panel) show that at low levels of *IP*, slightly higher N_liceA_ counts were observed in the coldest water. At the moderate water *T* (10 °C), N_liceA_ levels rose gradually with increasing levels of *IP*. However, in warmer water (13 °C), N_liceA_ levels rose rapidly with increasing *IP*. Captured fish were generally smaller in warm water (> 11 °C) suggesting that the largest fish had left the area as the water *T* increased (Additional file 1: Figure S4).

**Table 2:** Final zero-inflated negative binomial model of the effects of temperature (T), infestation pressure (IP) and their interaction on NliceA (data set 1)

	Coefficient	SE	*P* > *Z*	95% CI
Negative binomial portion of model				
*T*	-0.166	0.093	0.073	-0.348–0.016
*IP*	0.909	0.105	0.0001	0.703–1.114
T*IP interaction	0.803	0.096	0.0001	0.614–0.991
*IP*^2^	-0.545	0.087	0.0001	-0.716– -0.374
Intercept	2.960	0.079	0.0001	2.805–3.115
Alpha	1.074	0.111		0.877–1.315
Logistic (inflation) portion of model				
*IP*	-1.016	0.195	0.0001	-1.398– -0.634
Intercept	-2.006	0.239	0.0001	-2.473– -1.538

*Abbreviations*: *CI* confidence interval, *SE* standard error

#### Mediation analyses

The results from the mediation analysis with *T* as exposure of interest and *IP* (both linear and quadratic terms) as mediators and accounting for the significant T*IP interaction are shown in Table 3. Specific interpretations of the pure and direct estimates are also presented in Table 3. The direct effects are much smaller, suggesting that nearly all of the effects of *T* are mediated through its effect on *IP*. The estimated partition of the total effect of *T* that was mediated through *IP* was 78%, indicating that *T* increased louse counts primarily by raising the *IP*.

**Table 3:** Decomposition of effects from mediation analysis for the effects of temperature on N_liceA_std_ that are direct or mediated through *IP*, taking into account interaction between *T* and *IP*

Effect	Estimate	SE	Interpretation
Pure direct effect	0.007	0.060	The increase in log(N_liceA_) brought about by a 1 SD increase in *T* with *IP* held constant at an average *T*
Total direct effect	0.156	0.051**	The increase in log(N_liceA_) brought about by a 1 SD increase in *T* with *IP* held constant at the level resulting from that 1 SD increase in *T*
Pure indirect effect	0.418	0.047***	The increase in log(N_liceA_) brought about by increasing *IP* by the amount that would result from a 1 SD increase in *T* while holding *T* constant at an average *T*
Total indirect effect	0.567	0.073***	The increase in log(N_liceA_) brought about by increasing *IP* by an amount that would result from 1 SD increase in *T* while holding *T* constant at the level resulting from that 1 SD increase in *T*
% of total effect mediated	0.784		Based on a total effect of 0.723

*P < 0.05, **P < 0.01, ***P < 0.001

*Abbreviations*: IP, infestation pressure from fish farms; SE, standard error; SD, standard deviation; T, temperature

For the mediation analyses with *Year* as the exposure of interest and *IP* as the mediator, the overall estimate was 63%, indicating that most of the annual effects were mediated through *IP.* The variation in % mediated through *IP* varied from 42% to 100% between years, with some years having very large standard errors. No evidence existed of the interaction between *Year* and *IP*.

For the mediation analyses, with *Year* as the exposure of interest and *T* as the mediator, the overall estimate was 44%. The variation in % mediated through *T* varied from 3% to 36% between years. This indicates that more of the annual effects were mediated through *IP* rather than through changes in *T* from year to year. No evidence was observed of an interaction between *Year* and *T*.

### Dataset 1: Mobile (adult) louse counts

#### Descriptive statistics

**Fig. 5:**
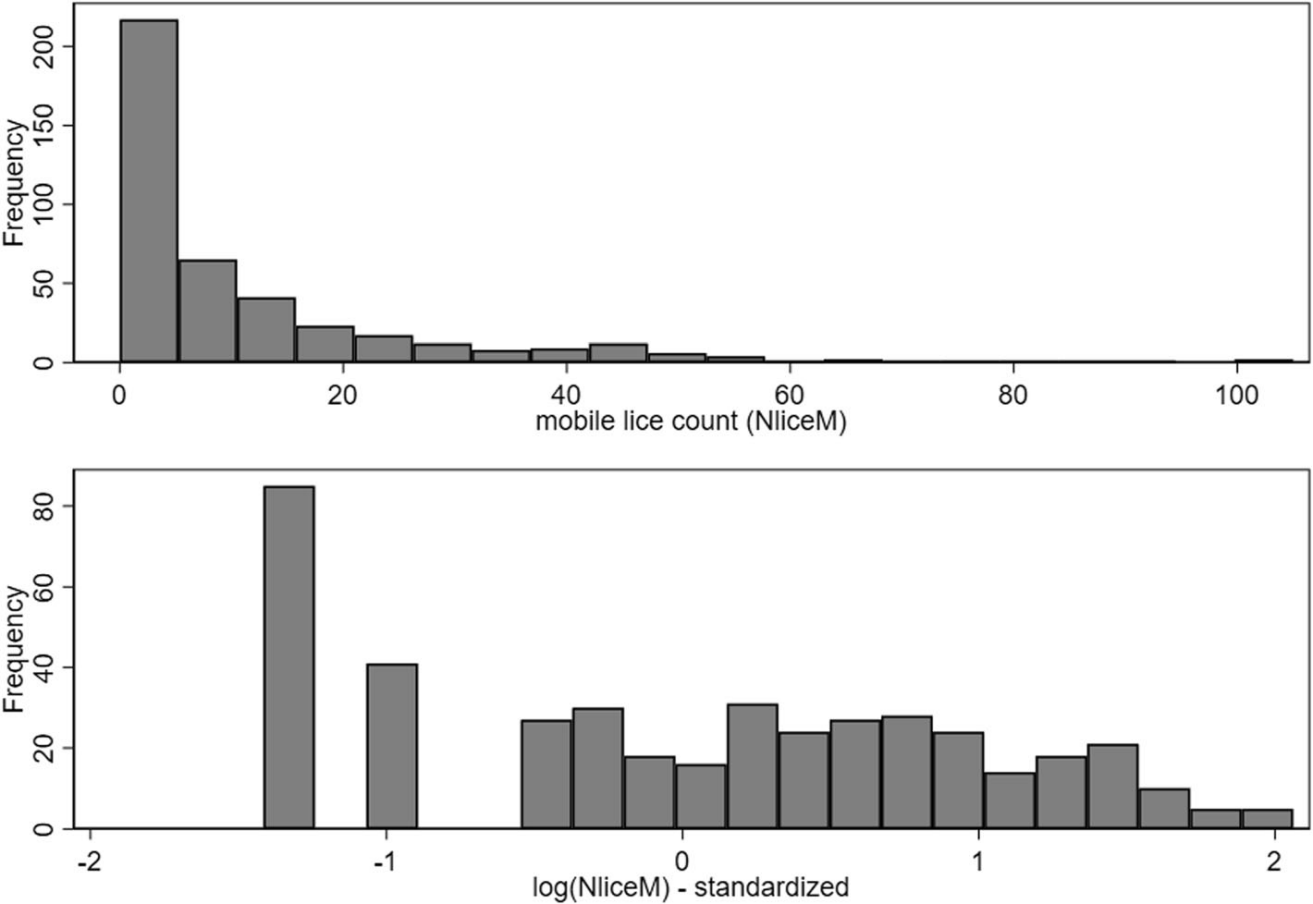
Histogram of adult louse counts - original (N_liceM_) and standardized (N_liceM_std_) versions - in Dataset 1

Histograms of N_liceM_ and log(N_liceM_) are shown in Fig. 5. The Lowess smoothed curves of all the relations among the standardized versions of continuous variables (including the T*IP interaction) are shown in Additional file 1: Figure S5.

#### Negative binomial models

Both *T* and *IP* had significant quadratic effects on N_liceM_, so the quadratic terms were retained in all subsequent models. No evidence was observed of an interaction between *T* and *IP*. The inclusion of *Year* in the negative binomial models did not have strong detrimental effects on the estimates of effects of *IP,* so *Year* was added as a random effect to the NB models. No evidence existed of zero inflation, so standard NB models were used, and the final model is presented in Table 4, with marginal predicted values for a range of *IP* values across three *T* ranges being shown in Fig. 4 (right panel).

**Table 4:** Final negative binomial model of the effects of *T* and *IP* on N_liceM_

	Coefficient	SE	*P* > *Z*	95% CI
*T*	-0.46	0.11	0	-0.67– -0.25
*T* ^2^	-0.21	0.08	0.01	-0.36– -0.05
*IP*	0.63	0.09	0	0.45–0.81
*IP* ^2^	0.19	0.07	0.01	0.05–0.33
Intercept	2.29	0.09	0	2.12–2.47
Alpha	1.61	0.12		1.39–1.87

*Abbreviations*: CI, confidence interval; SE, standard error

The most surprising difference in the results for N_liceM_ was that the effect of *T* was negative across all levels of exposure. Higher counts of mobile lice were observed at lower temperatures. However, regardless of *T,* increasing levels of *IP* were associated with increasing mobile louse counts. The estimate of alpha was 1.61 (95% CI: 1.39–1.87) indicating that the variance in N_liceA_ was much greater than would have been expected under a Poisson distribution.

#### Mediation analyses

Results from the mediation analysis of *T* as the exposure of interest and *IP* as the mediating variable are shown in Table 5. The direct effect was negative (lower N_liceM_ as *T* went up), but the indirect effect was positive (higher *T* resulted in higher *IP* which in turn raised N_liceM_). Consequently, the total effect was close to zero, and computation of a % mediated value would be meaningless.

**Table 5:** Decomposition of effects from mediation analysis for the effects of *T* on N_liceM_std_ that are direct or mediated through *IP*

Effect	Estimate	SE	Interpretation	
Natural direct effect	-0.43	0.054***	The increase in log(N_liceM_) brought about by a 1 SD increase in *T* with *IP* held constant at an average *T*	
Natural indirect effect	0.34	0.038***	The increase in log(N_liceM_) brought about by increasing *IP* by the amount that would result from a 1 SD increase in *T* while holding *T* constant at an average *T*	
Total effect	-0.1	0.046*	The increase in log(N_liceM_) brought about by increasing *T* by 1 SD	
% mediated	na			

*Abbreviations*: *IP* infestation pressure from fish farms, *na* not available, *SE* standard error, *SD* standard deviation, *T* temperature

*P < 0.05, ***P < 0.001

For the mediation analyses with *Year* as the exposure of interest and either *IP* or *T* as the mediators, the direct effects for individual years were all positive, and most were statistically significant, but indirect effects were all very small (some negative, some positive), and none were statistically significant. Overall estimates of % mediated were +0.03 and -0.03 for *IP* and *T,* respectively. This indicates that the annual effects on mobile (adult) louse counts were direct and due to factors other than the effect of *Year* on *IP* or *T*.

### Dataset 2: Attached and adult (mobile) louse counts (N_liceA_ and N_liceM_)

#### Descriptive statistics

Data were available from 1051 fish that were sampled between day 95 (6 April) and 214 (3 August) in the years 2009–2016. Attached louse counts ranged from 0 to 770, and adult louse counts ranged from 0 to 249 (Table 1). Histograms of the counts of attached and mobile lice are presented in Additional file 1: Figures S6 and S7. For both N_liceA_ and N_liceM_, no evidence of non-linearity was observed in the effect of day, so these values were included as a simple linear term in all models.

#### Negative binomial models

**Fig. 6:**
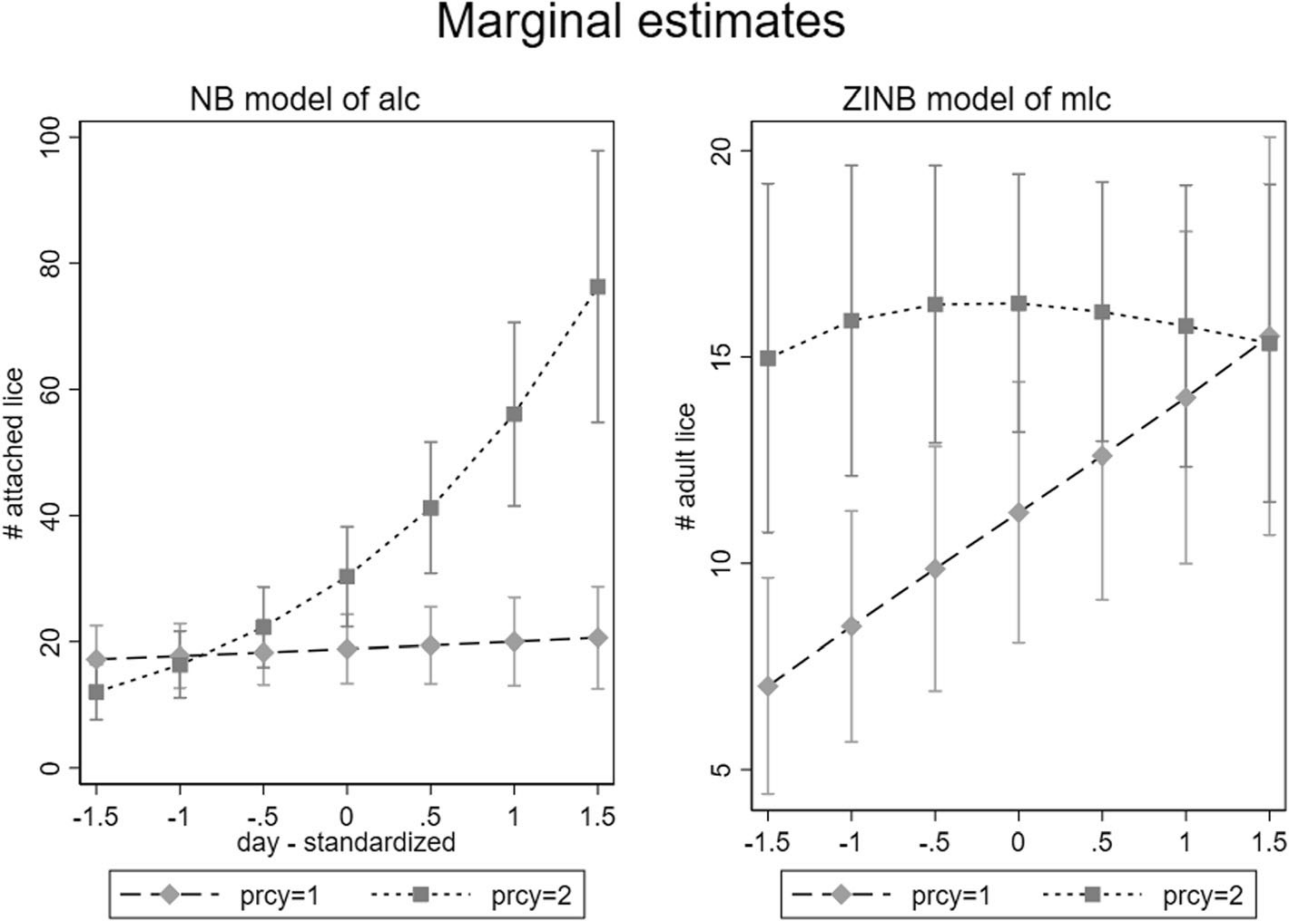
Predicted values from a negative binomial model of N_liceA_ (left panel) and a zero-inflated negative binomial model of N_liceM_ (right panel) from Dataset 2. Lines represent the predicted values in years in which the regional aquaculture operations were in their first or second production cycle year (prcy)

For attached lice, the ZINB model was not significantly better than the NB model, so the simpler standard NB model was retained. The final model is presented in Table 6. Marginal effect estimates by day, for both the first and second years of the production are presented in the left panel of Fig. 6. Time of year (*Day*) had no effect on attached louse counts during the first year of the production cycle (when louse counts were generally low), but counts increased dramatically towards the end of the second year.

**Table 6:** Final negative binomial model of the effects of production cylce year (*PC*), day and their interaction on attached lice counts (N_liceA_) (Dataset 2). 2016 omitted due to collinearity

	Coefficient	SE	*Z*	*P* > *Z*	95% CI
First *PC*	2.15	0.16	13.36	0	1.83–2.46
Day	0.06	0.07	0.88	0.38	-0.07–0.19
PC*Day	0.56	0.09	5.85	0	0.37–0.74
Year					
2010	1.49	0.14	-10.5	0	-1.77– -1.21
2011	0.22	0.2	1.1	0.27	-0.17–0.62
2012	0.21	0.15	-1.43	0.15	-0.51–0.08
2013	0.04	0.21	0.19	0.85	-0.37–0.45
2014	0.14	0.16	0.83	0.41	-0.19–0.46
2015	1.81	0.2	8.91	0	1.41–2.2
Intercept	1.13	0.13	8.6	0	0.87–1.39

*Abbreviations*: CI, confidence interval; SE, standard error

For mobile lice, the ZINB model was significantly better than the NB model, so it was retained. The final model is presented in Table 7. Marginal effect estimates by day, for both the first and second year of production, are presented in the right panel of Fig. 6. During the first year of the production cycle, a steady increase was observed in the number of adult lice. However, in year 2, virtually no change occurred in the number from day 95 to 214.

**Table 7:** Final zero-inflated negative binomial model of the effects of *PC*. day and their interaction on adult lice counts (N_liceM_) (Dataset 2). 2016 omitted due to collinearity

	Coefficient	SE	*Z*	*P* > *Z*	95% CI
Negative binomial portion of model					
First *PC*	1.62	0.16	10.34	0	1.31–1.93
Day	0.19	0.07	2.78	0.01	0.05–0.32
First PC*Day	-0.26	0.09	-2.82	0.01	-0.43– -0.08
Year					
2010	-0.71	0.13	-5.62	0	-0.96– -0.47
2011	0.53	0.19	2.87	0	0.17–0.9
2012	-0.98	0.14	-6.96	0	-1.26– -0.71
2013	0.06	0.21	0.26	0.79	-0.37–0.48
2014	-1.71	0.15	-11.62	0	-1.99– -1.42
2015	0.33	0.2	1.68	0.09	-0.06–0.72
Intercept	1.94	0.14	14.31	0	1.67–2.2
Alpha	1.4	0.1			1.23–1.6
Logistic (inflation) portion of model					
Day	-1.08	0.23	-4.68	0	-1.53– -0.63
Intercept	-2.92	0.38	-7.73	0	-3.66– -2.18

*Abbreviations*: CI, confidence interval; SE, standard error

## Discussion

This study demonstrates that the *IP* from fish farms plays an important role in the epidemiology of sea lice on wild sea trout. This result is indicated in the analysis using modelled *IP* from fish farms (Dataset 1) and using day of season and production cycle as a surrogate for modelled *IP* (Dataset 2). The data indicate that the recruitment of sea lice on wild sea trout (i.e. attached stages of lice) increases dramatically throughout the season when fish farms are in the second year of production; this can be explained by the observed interaction between the increasing *IP* and increasing *T*. Consequently, the effects of temperature alone cannot explain episodes of a high number of lice in the attached stages on sea trout in areas with high farming activity throughout Norway [14]. In fact, almost all of the observed effects of *T* on the attached louse counts on wild sea trout occur because *T* increases the *IP* from the fish farms (i.e. *T* effect is mediated through *IP*). However, no evidence exists for the adult mobile stages, which is not strongly linked to *IP* and surprisingly has a negative relation with *T*. In the following sections, we discuss the patterns observed on attached and mobile lice on sea trout separately.

### Attached sea lice on sea trout

During periods where *T* was high, the number of attached lice on sea trout increased exponentially with *IP*. During periods of colder water, no effect of *IP* was observed. These results corroborate the hypothesis that sea lice from fish farms play an important role in the infestation levels of sea lice on wild sea trout [16, 30, 31, 42], at least during periods when the seawater temperature is high. As argued by Jansen et al. [1], *T* and *IP* do correlate; in fact, *IP* is a function of temperature biologically, and in this instance, mathematically. *IP* is calculated based on louse numbers on farmed fish, biomass and temperature [25]. In an attempt to disentangle the association between *T* and *IP,* we applied a mediation analysis and provide evidence that the largest effect of *T* on the number of lice attached to sea trout is through the effect of *IP* from fish farms. This means that, in our study area, most of the effect of *T* on the observed louse counts of wild fish were most likely driven by increasing the processes of reproduction of lice on fish farms and the subsequent development and dispersal to wild fish. Both Helland et al. [30] and Serra-Llinares et al. [31] found that temperature was an important additional predictor in their model of sea lice on sea trout. Similar to what we observed in our study, Serra-Llinares et al. [31] also found an interaction between temperature and *IP*, which suggested that the slope and intercept of the association with *IP* were steeper and higher when the temperature was warmer. This is very similar to our results. However, their model also included 16 parameters (counting both the zero-inflation and count part of the model), making the comparison with our model somewhat convoluted. Furthermore, the estimation of *IP* in their studies and our study are somewhat different, making the direct comparison even more complicated. Even so, the general message is similar: temperature does increase the natural infestation levels on wild sea trout, but these levels will increase much more dramatically with high *IP* from surrounding fish farms. In addition, our study goes further by demonstrating that the effect of temperature is mainly mediated through *IP*.

The link between the *IP* model and recruitment of the attached stages of lice on sea trout corroborates the findings of Kristoffersen et al. [43], who demonstrated that the *IP* used in their study could explain a large proportion (~39%) of the variation in lice abundance on salmon smolts placed in sentinel cages at different locations along the coast of Norway. The infestation levels on fish in sentinel cages are thought to be easier to predict than sea trout, as the spatial location of the fish is known throughout the period of infestation compared to sea trout where the exact location of infestation is unknown. Even so, our model does suggest that the *IP* is also an important predictor of the attached lice on sea trout, corroborating these results. However, the infestation levels on wild sea trout are much higher than what has been observed on caged salmon, which begs the question of whether the infestation on sentinel cages correctly mimics the way sea trout encounters lice in the wild. However, because we do not know how long the wild-caught sea trout had been exposed to the infestation pressure, we found it difficult to compare the two.

Approximately 60% of the between-year variation in the attached louse counts on sea trout was mediated through *IP*, whereas ~40% of the yearly variation was mediated through *T*. However, importantly, these values are not additive (they are not independent), so that the total effect of *Year* mediated through *T* and *IP* is unknown (but would be > 60% and < 100%). This indicates that additional unknown factors exist, which may vary from year to year and have an effect on the recruitment of sea lice on wild sea trout. One such mechanism is wild fish to wild fish infestations. For example, mobile stages of lice on sea trout are relatively high throughout the season in years when surrounding fish farms are in their second year of production (see results from mobile lice). Sea trout are known to stay close to shore [44], and most likely aggregate in areas of high prey abundance. The lice from individuals with high infestation levels of adults may consequently produce infective stages of sea lice in key sea trout habitat, particularly when the temperature is high. In addition, outbreaks with high louse counts do occasionally occur in areas without fish farms (e.g. recently documented in the Norwegian surveillance programme of sea lice in 2017), and the wild fish to wild fish infestation should not be ignored when trying to understand the sea lice population dynamics. Furthermore, we had no data on salinity or freshwater input in our system. Helland et al. [30] found that freshwater is an important predictor of louse numbers on sea trout. Shephard et al. [16] also found that rainfall interacts with distance from fish farms in a model predicting the size of the sea trout, suggesting that sea lice from fish farms impact the growth of young sea trout more during dry years. Consequently, the unexplained year-to-year variation may be due partially to differences in available freshwater and salinity the different years.

The results from the model including *IP* were also corroborated by the results from the model using the production cycle of the fish farms and the day of the season as a predictor variable for the entire sampling period, which clearly demonstrated that the numbers of attached lice on sea trout only increased in years where surrounding fish farms were in second year of production, but not in years when fish farms were in the first year of production. Biennial patterns of high abundance of sea lice on wild fish and in plankton due to fallowing cycles have been reported in various other studies [22, 26–28] and, in general, serves as strong evidence that the presence of fish farms affects the parasite dynamics of sea lice on wild salmonids. Furthermore, the clear seasonal pattern with increasing infestation later in the season in years when fish farms are in the second year of production demonstrates that sampling needs to consider the seasonal progression of infestation levels on fish farms when samples are collected.

### Mobile lice on sea trout

The effects of the *IP*, *T* and production cycle on adult lice (N_liceM_) were perhaps not as intuitive as the pattern observed on the attached lice. The effect of *IP* from fish farms was as expected, positive. The effect of *T*, however, was negative. This unexpected result indicates that fish sampled in periods when *T* was lower had a relatively higher adult lice count than what was expected according to the *IP*. This may be because the adult lice, to a larger extent, reflect an *IP* over a prolonged period of time because the lice can survive on sea trout as adults for months. This could explain the observed effect in the second dataset looking at production cycle and day of season, which suggests that, during the first year of production, adult louse numbers increase throughout the season, whereas during the second year, the adult louse numbers stay high throughout. This may indicate that the sea trout had already experienced a cumulative high *IP* in the preceding months. Mobile lice can directly impact the physiology [10] and, consequently, the behaviour of sea trout [45]. When *T* increases, the severity of these effects may increase, and the sea trout with the highest infestations of mobile sea lice may return to freshwater or die, leaving a population with lower louse counts when the water is warm. This could be the reason why we see a decreasing size of captured sea trout with increasing temperature (Additional file 1: Figure S4). Sea trout are known to respond to lice-induced or other factors causing osmoregulatory stress by moving to brackish water or freshwater in order to restore the osmotic balance [44]. This phenomenon has been termed ‘premature return’ [46, 47], although this term perhaps is not adequate because it describes an adaptive behaviour to physiological stress, which can also happen under natural conditions. However, when the mechanism behind this behaviour is the *IP* from fish farms, ‘premature return’ does serve as a description of a behaviour that would not have occurred if fish farms were not present. Consequently, ‘premature return’ can be seen as a manmade effect that hinders sea trout to exploit the marine habitat naturally [45]. Studies on the effect of sea lice on behaviour have been conducted using acoustic tags and an anti-parasitic agent in the Hardangerfjord [15, 48]. Results from these studies, however, are ambiguous, as they demonstrate that anti-parasitic agents do not increase survival, but that years with high *IP* lead to higher likelihood of sea trout staying in freshwater for a longer period of time. Exactly how *T* will impact this pattern is unclear and warrants further studies.

If the sea trout were unable to rid themselves of the high sea louse levels that were observed on the sea trout every second year (2010, 2012, 2014 and 2016), the infestation would lead to direct mortality for a large proportion of the population. More than 60% had more than 0.3 louse/gram at the end of June in our samples, and this level has been considered a lethal dose of sea lice for sea trout in some publications [10, 37, 49]. However, even fish with heavy burdens of lice can survive by returning to freshwater for a period before returning to sea [46]. The proportion that survives and the cost of sea louse infestation on sea trout is therefore not necessarily proportional to the abundance of the lice on fish but is a complex function of the infestation levels and the physical environment (i.e. temperature, salinity and distance to a freshwater source). A recent study has also explored the effect of sea lice on the vertical behaviour of trout, demonstrating that fish with more lice spend more time at the surface, where salinity is low, and can lose substantial amounts of lice by doing so (A. Mohn, MSc thesis, UiB). On the other hand, the predation risk is likely to increase as the trout changes its behaviour, and this behaviour could make the trout more conspicuous to both fish and avian predators. For management, this creates a complexity that makes it difficult to directly link sea louse counts to threshold levels of mortality observed in the laboratory.

## Conclusions

When looking at the recently recruited sea lice on sea trout (i.e. attached sea lice), the effects of temperature (*T*) and infection pressure (*IP*) interacted, resulting in large increases in louse counts when the water was warm. Nearly all of the effects of rising *T* were mediated through *IP*. Approximately half of the year-to-year variation in the attached louse counts was explained by changes in *IP*, but other (unmeasured) factors accounted for between 25 and 50% of the annual variation. Attached louse counts remained low when neighbouring farms were in the first year of the production cycle but rose substantially throughout the season in the second year. In contrast to attached lice, higher counts of mobile lice were generally seen at lower water temperatures, but this difference may have been attributable to more heavily infested fish being removed from the population. Temperature had an indirect positive effect on mobile louse counts by increasing the *IP*, which in turn raised the mobile louse counts. Mobile louse counts rose steadily during the year when neighbouring farms were in the first year of the production cycle and stayed high throughout the second year. In conclusion, the estimates of the *IP* effect on louse counts, along with a clear biennial pattern emerging due to the production cycle of fish farms, clearly indicate that fish farms play an important role in the parasite infestations on wild sea trout.

## Additional file

Additional file 1: **Text 1.** Removal of *Year* as potential confounder**. Table S1.** Correlations between day and infection pressure (*IP*) in data set 1. **Table S2.** Comparison of effect estimates from a negative binomial model of mobile lice counts (N_liceM)_ and a linear model of counts log-transformed and standardized (N_liceM_std_) (424 observations in each model). **Figure S1.** Plot of coefficients for estimated infection pressure (*IP*) when evaluated for individual years. **Figure S2.** Scatterplots and lowess smoothed curves showing the relationships between *Day* and estimated infection pressure (*IP*), broken down by year of the production cycle in regional aquaculture operations. Based on Dataset 1. **Figure S3.** Lowess curves for evaluating linearity of relationships among continuous variables included in the analysis of attached lice counts (N_liceA_) in Dataset 1. **Figure S4.** Scatterplot and lowess smoothed curve of the relationship between trout length and water temperature at the time of sampling. **Figure S5.** Lowess curves for evaluating linearity of relationships among continuous variables included in the analysis of mobile lice counts (N_liceM_) in Dataset 1. **Figure S6.** Histogram of attached lice counts (N_liceA_) and log(N_liceA_) in Dataset 2. **Figure S7.** Histogram of adult (mobile) lice counts (N_liceM_) and log(N_liceM_) in Dataset 2. (DOCX 2858 kb)

## Abbreviations

N_lice_: Number of lice on individual sea trout
W: Weight in grams of fish
PC: Production cycle
DY: Day of year
A: Area
*IP*: Infestation pressure
*T*: Estimated temperature
