# Supplementary material for: Parasitic sea louse infestations on wild sea trout: separating the roles of fish farms and temperature
Source: Parasit Vectors. 2018 Nov 29;11:609. doi: 10.1186/s13071-018-3189-6 (PMC6267784; doi:10.1186/s13071-018-3189-6)
Supplement: Supplementary file 1 — Text 1. Removal of Year as potential confounder. Table S1. Correlations between day and infection pressure (IP) in data set 1. Table S2. Comparison of effect estimates from a negative binomial model of mobile lice counts (NliceM) and a linear model of counts log-transformed and standardized (NliceM_std) (424 observations in each model). Figure S1. Plot of coefficients for estimated infection pressure (IP) when evaluated for individual years. Figure S2. Scatterplots and lowess smoothed curves showing the relationships between Day and estimated infection pressure (IP), broken down by year of the production cycle in regional aquaculture operations. Based on Dataset 1. Figure S3. Lowess curves for evaluating linearity of relationships among continuous variables included in the analysis of attached lice counts (NliceA) in Dataset 1. Figure S4. Scatterplot and lowess smoothed curve of the relationship between trout length and water temperature at the time of sampling. Figure S5. Lowess curves for evaluating linearity of relationships among continuous variables included in the analysis of mobile lice counts (NliceM) in Dataset 1. Figure S6. Histogram of attached lice counts (NliceA) and log(NliceA) in Dataset 2. Figure S7. Histogram of adult (mobile) lice counts (NliceM) and log(NliceM) in Dataset 2. (DOCX 2858 kb) [file 13071_2018_3189_MOESM1_ESM.docx]

# Additional file 1

**Text 1.** Removal of year as potential confounder

It would be normal practice to include year as a potential confounder in many studies. While “year” is not a biological cause of louse counts, it may be a surrogate for factors which might influence both IP and louse counts, and hence might be a confounder. However, in our data set 1, “year” was actually a surrogate for IP because of the very strong association between year and IP (R^2^=64%). In essence, we had two measures of IP – the direct value and “year” as a surrogate. We could only include one in the model and the direct measure was what we were primarily interested in. To determine if omitting the year as a confounder had an adverse effect on the negative binomial (NB) models of N_liceA_ with *IP* as a predictor (described below), separate NB models were fitted for each year for which data were available. Given the limited data available for each year it was necessary to keep these models relatively simple so only the linear function of IP was included. No estimate was obtained for 2016 because of the small number of observations (n=46) and the fact that there was very little variation in IP for those 46 fish. A plot of the annual IP coefficients is shown in Figure S4 along with an overall estimate of the IP effect derived from a model without year included. While there was considerable variation among the point estimates, all of the confidence intervals for the annual values encompassed the overall estimate (β = 0.69) suggesting that not including year in the models was not detrimental to the estimates of IP effects.

**Text 2.** Using linear versus negative binomial models in mediation analysis

Software for mediation analyses with NB or ZINB distributed outcome variables is not currently available. Consequently, the suitability of using linear models with log(N_liceA_) as outcome was explored by comparing ZINB and linear models in terms of: their estimated coefficients (both models have coefficients on the log scale) (Table S2). The estimates were quite similar. The correlations between the observed and predicted values from each of the two models were also very close. Both the NB and ZINB models had correlations of 0.43 on the count scale and 0.57 and 0.57 respectively on the log(count) scale. This provided evidence that a mediation analyses utilising linear models of log(N_liceA_) were acceptable. The results were similar when comparing models for log(N_liceM_).

**Table S1.** Correlations between day and infection pressure (*IP*) in data set 1

| year | # of observations | correlation |
| --- | --- | --- |
| 2012 | 110 | 92% |
| 2013 | 80 | 94% |
| 2014 | 101 | 93% |
| 2015 | 87 | 85% |
| 2016 | 46 | 98% |

**Table S2** Estimates of effects of *T* and *IP* on from a ZINB of N_liceA_ and linear model of N_liceA_std_

| **Variable** | **Zero-inflated negative binomial** | **Linear model of log(N_liceA_)** |
| --- | --- | --- |
| *T* | -0.206* | -0.103 |
| *IP* | 1.0866*** | 1.084*** |
| *IP*^2^ | -0.662*** | -0.430*** |
| *T*IP* interaction | 0.815*** | 0.688*** |
| Intercept | 2.871*** | 1.921*** |

**P* < 0.05, ***P* < 0.01, ****P* < 0.001

**Table S2.** Comparison of effect estimates from a negative binomial model of mobile lice counts (N_liceM)_ and a linear model of counts log-transformed and standardized (N_liceM_std_). (424 observations in each model)

| Variable | NB model | Linear model |
| --- | --- | --- |
| *T* | -.473*** | -.492*** |
| *T^2^* | -160 | -.195* |
| *IP* | .626*** | .800*** |
| *IP^2^* | 170* | .236*** |
| *intercept* | 2.252*** | 1.443*** |

**P* < 0.05, ***P* < 0.01, ****P* < 0.001

**Figure S1.** Plot of coefficients for estimated infection pressure (*IP*) when evaluated for individual years (top 4 lines) and overall (bottom line). *Note*: insufficient variation existed in the *IP* in 2016 to provide a valid estimate of the coefficient

**Figure S2.** Scatterplots and lowess smoothed curves showing the relationships between *day* and estimated infection pressure (*IP*), broken down by year of the production cycle in regional aquaculture operations. Based on dataset 1.

**Figure S3.** Lowess curves for evaluating linearity of relationships among continuous variables included in the analysis of attached lice counts (N_liceA_) in dataset 1

.

**Figure S4.** Scatterplot and lowess smoothed curve of the relationship between trout length and water temperature at the time of sampling.

**Figure S5.** Lowess curves for evaluating linearity of relationships among continuous variables included in the analysis of mobile lice counts (N_liceM_) in dataset 1

**Figure S6.** Histogram of attached lice counts (N_liceA_) and log(N_liceA_) in dataset 2

**Figure S7.** Histogram of adult (mobile) lice counts (N_liceM_) and log(N_liceM_) in dataset 2
